# Editorial: Phosphoinositides and their phosphatases: Linking electrical and chemical signals in biological processes

**DOI:** 10.3389/fphar.2015.00142

**Published:** 2015-07-08

**Authors:** Susy C. Kohout, Carlos A. Villalba-Galea

**Affiliations:** ^1^Department of Cell Biology and Neuroscience, Montana State UniversityBozeman, MT, USA; ^2^Department of Physiology and Biophysics, Virginia Commonwealth University School of MedicineRichmond, VA, USA

**Keywords:** voltage-sensing phosphatase, membrane potential, cell signaling, phosphatidylinositols, pten

The voltage-sensing phosphatase (VSP) has changed the way we think about both cellular electrical activity and PIPs (phosphatidylinositol phosphates). Originally discovered in 1999 (Chen et al., 1999), these proteins were not recognized as electrically-controlled enzymes until 2005 (Murata et al., 2005). They constitute the first, and so far the only, example of an enzyme linking electrical signals at the plasma membrane to the catalysis of PIPs (Murata et al., 2005), a ubiquitous family of intracellular signaling molecules (Di Paolo and De Camilli, 2006; Balla, 2013). Before the discovery of VSP, there were no known direct links between the two. Textbook examples would represent this connection with arrows, alluding to indirect or “yet-to-be-defined” signaling pathways. Now we know that VSP serves as a direct connection between the electrical nature of the cell and PIPs, lipid second messengers that are critical for cell survival. However, many questions remain unanswered regarding VSP and its electrical regulation of cellular processes.

With the discovery of VSP, the membrane potential must now be considered when studying PIP regulators. PIPs are involved in almost all aspects of cell physiology from survival, proliferation, and migration to pre-programed cell death (Di Paolo and De Camilli, 2006; Logothetis et al., 2010; Koch and Holt, 2012; Balla, 2013). For example, PIP concentrations are actively polarized in migrating cells with phosphatidylinositol-3,4,5-trisphosphate (PI(3,4,5)P_3_) on the leading edge and phosphatidylinositol-4,5-bisphosphate (PI(4,5)P_2_) on the lagging edge (Leslie et al., 2008). These gradients in the concentration of PIPs are necessary for activation of Rac and Rho leading to cell motion. PIPs are also crucial for cell growth: PI(3,4,5)P_3_ activates the mTor cascade leading to increased protein, membrane, and nucleic acid production (Dibble and Manning, 2013). Many human diseases have been associated with altered homeostasis of PIPs, including cancer, developmental disorders, and Alzheimer's disease (Simpson and Parsons, 2001; McCrea and De Camilli, 2009; Hakim et al., 2012). Though the physiological relevance of VSP is not yet defined, it is still crucial to human health to understand how PIPs are regulated and that now includes VSP.

All cells have an asymmetric composition of ions across their plasma membrane, which, combined with selective permeabilities for these ions, results in a difference in the electrical potential across their plasma membrane. This difference, called the membrane potential, constitutes a form of cell signaling and a source of energy, both driving many biological processes. This electrical potential difference powers neuronal excitability as well as more general processes like proliferation, migration, and development (Levin, 2007; Sundelacruz et al., 2009; Yao et al., 2011). Regulation by the membrane potential has long been the sole purview of ion channels and transporters and that has influenced what questions are asked regarding the changing potential. With our new knowledge of VSP, the changing membrane potential can directly signal the cell by modulating mTor and cell growth pathways, leading to abnormal growth or the M-current in sympathetic ganglion, leading to hyperexcitability.

The articles in this Special Topic highlight several features of VSP including its unique activation, its similarities to other enzymes and its use as a versatile tool to study other proteins. In the review article by Hobiger and Friedrich (2015, p. 20), the authors compare the structural similarities and differences between the broader family of protein tyrosine phosphatases and one of its newest members, VSP. They suggest a catalytic mechanism based on this comparison. Castle et al. (2015, p. 63) investigate the activation mechanism of VSP by probing the C2 domain, the C-terminal domain of VSP that has been largely unrecognized before the recent crystal structures showed a direct contribution of the C2 residue Y522 into the active site. The work by Mavrantoni et al. (2015, p. 68) explores the techniques that are used to test VSP and address some of their limitations including the need for expensive electrophysiology equipment as well as the limitations of using channels as functional reporters. They take their methods and apply them to a chimera between the *Ciona intestinalis* VSP and human PTEN and show how the chimera allows for the investigation of PTEN using standard techniques but with the advantage of regulated activation, voltage.

Beyond the molecular mechanism underlying VSP activity, Mori et al. (2015, p. 22) review the use of VSP as a relatively simple tool for manipulating PI(4,5)P_2_ concentrations in cells. They have used VSP to study the PI(4,5)P_2_ regulation of transient receptor potential canonical channels involved in receptor-operated calcium currents. Along the same lines, Rjasanow et al. (2015, p. 127) use VSP as a tool that gives them precise control over the PI(4,5)P_2_ concentrations in the membrane. These authors compared the relative PIP affinities between several ion channels. They also point out an important limitation that the channels must already have a known specificity for a particular PIP because VSP does not destroy PIPs in contrast to phospholipase C; instead, it generates multiple PIPs. All together, these articles underscore the features of VSP and expand our understanding of its function and utility.

Though VSP remains relatively unknown to many, this nascent field has shown fast initial growth. The unique nature of these enzymes has inspired many to investigate their properties as well as take advantage of them. Many questions remain unanswered regarding VSP such as how the voltage sensor couples to the enzyme and whether the phosphatase domain is brought to the membrane for activation or whether a conformational change within the active site determines activation. We look forward to the studies that will address these and the many other questions that persist in this exciting field.

## Conflict of interest statement

The authors declare that the research was conducted in the absence of any commercial or financial relationships that could be construed as a potential conflict of interest.
